# Risk of *Bacillus cereus* in Relation to Rice and Derivatives

**DOI:** 10.3390/foods10020302

**Published:** 2021-02-02

**Authors:** Dolores Rodrigo, Cristina M. Rosell, Antonio Martinez

**Affiliations:** Instituto de Agroquimica y Tecnología de Alimentos (IATA-CSIC), Paterna, 46980 Valencia, Spain; crosell@iata.csic.es (C.M.R.); amartinez@iata.csic.es (A.M.)

**Keywords:** *Bacillus cereus*, rice, poisoning

## Abstract

Rice is a very popular food throughout the world and the basis of the diet of the citizens of many countries. It is used as a raw material for the preparation of many complex dishes in which different ingredients are involved. Rice, as a consequence of their cultivation, harvesting, and handling, is often contaminated with spores of *Bacillus cereus*, a ubiquitous microorganism found mainly in the soil. *B. cereus* can multiply under temperature conditions as low as 4 °C in foods that contain rice and have been cooked or subjected to treatments that do not produce commercial sterility. *B. cereus* produces diarrhoeal or emetic foodborne toxin when the consumer eats food in which a sufficient number of cells have grown. These circumstances mean that every year many outbreaks of intoxication or intestinal problems related to this microorganism are reported. This work is a review from the perspective of risk assessment of the risk posed by *B. cereus* to the health of consumers and of some control measures that can be used to mitigate such a risk.

## 1. Introduction

*Bacillus cereus* is present in many foods due to its ubiquitous nature and has become one of the top ten responsible for many cases of food and waterborne outbreaks in humans. The demands of consumers for complex and mildly processed foods with a limited refrigerated shelf-life are driving this increase on the *B. cereus* outbreaks all over the world as has been recognized by the European Food Safety Authority EFSA and Center for Disease Control (CDC) report [1]. In 2018, *B. cereus* was involved in a total of 98 reported outbreaks among members’ states of the European Union [1]. The impact of those numbers is clearer by saying that those represented the 1.9% of total outbreaks in the European Union, with 1539 people affected with 111 hospitalizations and 1 death [1]. Moreover, recent outbreaks in other countries have been also associated with this pathogen, e.g., 45 cases identified in an outbreak at a restaurant in Canberra (Australia) [2] and 200 students affected in an outbreak at a school in China [3]. Two types of food disease can be produced by *B. cereus*: diarrhoeal and emetic syndromes. Diarrhoeal illness is produced when enough *B. cereus* cells are consumed and the microorganism is implanted and grows in the small intestine producing the toxin, whereas the emetic syndrome appears when a food containing pre-formed cereulide toxin produced during *B. cereus* growth is consumed [4,5].

Generally, *B. cereus* has been associated with complex foodstuffs that include rice as ingredient; nevertheless, other rice-based products and farinaceous foods, such as pasta and noodles, can be also a vehicle for contamination and being involved in *B. cereus* intoxication [5]. This fact has promoted research on rice and carbohydrate-rich products and on improving decontamination and processing technologies that may reduce the risk of *B. cereus* poisoning. Outbreaks caused by *B. cereus* are due, in a large number of occasions, to the consumption of rice contaminated with spores or vegetative cells [6,7] (about 95% of cases of emetic disease are related to the consumption of rice [8]). Specifically, this etiological agent produced gastrointestinal diseases caused by the consumption of Chinese fried rice [9,10]. This is due to the way in which it is cooked; it is boiled in large quantities, kept unrefrigerated for several hours, depending on consumer’s demand, before being further processed (fried or heated). During this unrefrigerated storage the microorganism can grow and/or produce the emetic toxin that will not be destroyed or inactivated by a subsequent processing step [6]. These culinary practices are coherent from the logistical point of view; however, rice should be stored below 7 °C or above 63 °C, which are the limit temperatures for the germination of heat-resistant spores [11]. Those authors found that in 40 samples of local and imported rice, all of them contained *B. cereus* concentrations larger than 1100 CFU/g. Other authors [12] found that 94 out of 178 raw rice samples were contaminated with *B. cereus* in the United States. Therefore, reported results confirm how ubiquitous this microorganism, especially in rice-based foods, is. Nevertheless, the microbial loads are different depending on the rice form, being the presence of high concentrations of microorganisms more common in brown rice than in white rice, due to the processing that the cereal receives in each case. In fact, a study carried out with samples of white and husked rice reported concentrations of the pathogen around 2.5 × 10^1^ CFU/g in husked rice and 2.5 × 10^3^ CFU/g in brown rice [13].

Rice is the grain of herbaceous plants of the genus *Oryza* cultivated for more than 8000 years and of which about 750 million tons are produced annually mainly for human food, although the lower quality crops are destined to animal feeding [14]. It is a staple food for more than half of the world population and especially in underdeveloped countries. This cereal is presented to the consumer in different ways: whole, husked, or white depending on the treatment to which the grain was subjected. It also accepts many forms of industrial cooking and processing, steaming, parboiling, instant, ground (rice flour, pasta, and cookies) [15]. Each of them represents a different risk for the consumer depending on the subsequent treatment that consumer applies prior to consumption.

Rice, with a pH close to 7, consisting of 79% of carbohydrates, 7% protein, and 2% fat, plus vitamins and minerals, can act as an excellent growth medium for *B. cereus* once it has been cooked because it is in that moment when the humidity of the substrate reaches water activity values suitable for the growth of the microorganism. Even if the vegetative cells of *B. cereus* do not grow, they can survive 48 weeks on fresh and dry storage without loss of viability. Nevertheless, the viability of the pathogens is reduced after 16 weeks, if the storage occurs at temperatures above 45 °C with water activity around 0.78 [8].

The main problem posed by contamination with *B. cereus* of foods is the presence of heat-resistant spores that survive normal cooking temperatures for rice, or other raw materials or processed products, which is usually boiling water close to 100 °C [16]. Studies show that during normal cooking, around 20 min depending on the variety of rice, there are 2–3 decimal reductions on the initial spore load so the risk in the final product depends largely on the initial concentration of microorganisms and hygienic measures during handling, cooking, or processing [9,17]. After cooking, the remaining spores are capable of growing up to 10^7–^10^9^ CFU/g after 24 h at 26 or 32 °C respectively [10,11,18,19]. Spores germinate and grow depending on storage temperature; optimum growth temperatures in rice are 30–36 °C. After 10 days of storage at 8 °C, a growth of 10^4^ CFU/g to 10^8^ CFU/g was observed [20].

This review paper addresses the problem of *B. cereus* in rice and its derivatives under a microbiological risk assessment perspective, summarizing some control measures and improving processing technologies that could be considered to reduce the risk of the presence of the microorganism in these products and *B. cereus* toxin.

## 2. Hazard Description and Growth in Rice

*B. cereus* is a rod shaped spore-forming bacterium that belongs to the *Bacillus* genus. It is Gram-positive and motille due to flagels. *B. anthracis, B. cereus*, *B. mycoides, B. thuringiensis, B. pseudomycoides,* and *B. weihenstephanensis* are representative species of the 18 identified belonging to this genus [21]. Data of the genoma have shown that *B. anthracis, B. cereus,* and *B. thuringiensis* are very closely related; *B. thuringiensis* is an insect pathogen used in biocontrol and *B. anthracis* bacterium is responsible for anthrax [22,23]. These species show phenotypic properties and a high level of similarity in their DNA, making biochemical identification quite difficult [22]. Different species had traditionally been differentiated by their phenotypic characteristics (e.g., shape, optimal growth temperature, resistance to acidity) but currently, other more powerful methods are being used such as digital DNA-DNA hybridization (dDDH) and/or average nucleotide identity (ANI) values [24], signature sequences (e.g., in the 16 S rRNA and cspA genes) or the presence of specific virulence factors (e.g., cytK-1 or anthrax toxin genes), and MALDI-TOF MS analysis [25] or real time PCR [26]. A recent study for Carroll [27] describes the first whole genome sequencing (WGS) characterization of isolates linked to an outbreak caused by members of the *B. cereus* group.

*B. cereus* is a habitual saprophyte, which is resistant to low humidity, high temperatures, dehydration, radiation, and acidity; spores are ubiquitous in the environment, inhabitant of soil, water, vegetables, and air and can be found in the soil at concentrations in the order of 10^6^ CFU/g [28]. This microorganism is of interest in public health as it is considered an opportunistic pathogen that produces food toxins [16,29].

Within the *Bacillus* genus, *B. cereus* is the species most frequently associated with food outbreaks [30]. *B. cereus* is a facultative aerobic bacillus that can grow in highly variable conditions, a broad pH range between 4.5 to 9.5, at a minimum water activity for growth of 0.93, and in a broad range of temperatures from 4 °C (psychrotrophic strains) to 48 °C and at a NaCl concentrations up to 7% [31]. Although, as mentioned before, *B. cereus* tolerates a wide range of pH, the presence of 0.1% acetic acid is sufficient to inhibit the growth of the microorganism [29]. The optimum pH range from 6 to 7 and its tolerance to stress conditions due to pH, improves under anaerobically conditions [32]. Some strains of *B. cereus* are motile thanks to peritric flagella, although non-motile strains have also been described [13,33].

Although *B. cereus* have been considered as pathogenic microorganisms, researches described that some strains of this microorganism can be considered as beneficial and they have been used as animal probiotics in some formulations [34] and as growth promoters in plants [35].

The optimal growth temperature of *B. cereus ranges* between 30 and 40 °C although some strains can grow at 55 °C. Nevertheless, there are studies that have described that the strains responsible for emetic syndrome have a minimal growth temperature of 15 °C [31]. Even some strains show tolerance to lower temperatures, 4 °C, being considered psychrotrophic or psychrotolerant. Particularly, a study has suggested that *B. cereus* isolates from dairy products have adapted to those environmental conditions [36].

Vegetative cells die immediately below pH 4.3 in relation to previous exposure to acids in the environment before being ingested, despite the fact that some strains show great tolerance to gastric acids [37]. That could be the reason why the most frequent disease produced by the consumption of rice at neutral pH is the emetic syndrome, because at that pH, the microorganism lacks the necessary resistance against gastric acids to pass the intestinal tract and grows in the intestine producing toxins for emetic syndrome. By contrast, the spores have great tolerance to pH, being their viability from pH 1 to 9 [37]. In those conditions, when spores are ingested with the food, they can germinate in the small intestine and produce the diarrhoeal toxin.

Growth of *B. cereus* is optimal in the presence of oxygen, although the microorganism can grow anaerobically, but toxin production is undetectable in this environment [31]. Regarding the water activity (a_w_), for vegetative cells, it should be in the range of 0.912 to 0.950 for growth [38].

Rice derivatives (boiled, fried), due to its composition and chemical characteristics, represent an excellent growth medium for bacteria and can support the growth of *B. cereus* at different temperature conditions. *B. cereus* spores can survive perfectly in the dehydrated rice, without loss of viability for at least 48 weeks of storage. However, some loss of viability has been observed if it is stored at 45 °C and water activity of 0.78 [39].

*B. cereus* spores have the ability to survive the cooking treatments commonly given to rice. There is high heterogeneity in the thermal resistance of the spores. Some authors [40] indicated decimal reduction (D) values between 0.94 and 11 min at 95 °C and 0.22 and 2.5 min at 100 °C in double-distilled water. Another study reported a D value of approximately 3.5 min for spores in rice at 97.8 °C [41]. With those data, and considering a standard cooking process for rice, for example 100 °C for 20 min, it is clear that there will be some level of reduction in the number of spores in the food, but it will not be enough to ensure the food safety of the food for this microorganism.

The spores that remain alive after a long-term storage of cooked rice, even at low temperatures, germinate and some degree of growth of *B. cereus* will take place. Ultee et al. [42] indicated that *B. cereus* reached levels of 10^4^ to 10^8^ in ten days when the product was stored at 8 °C.

## 3. *B. cereus* Characterization Included Dose-Response Relationship

*B. cereus* produces two types of illness, the emetic and diarrhoeal, depending on the context at which it grows. The diarrheal syndrome is produced as a consequence of the ingestion of a large number of vegetative cells or spores that pass the stomach barrier, during their growth in the small intestine [43]. The emetic toxin “cereulide” is a cyclic peptide, produced during the growth of *B. cereus* in the food itself, when the conditions of pH, water activity, and temperature are suitable [44]. It has a strongly hydrophobic character, therefore, to cause food poisoning, it must be attached to the target cells attached or dissolved in vehicles found in food [44]. This type of food intoxication is most often associated with the ingestion of cereal products, especially rice [45,46].

Agata et al. [47] studied the growth and emetic toxin production of *B. cereus* in cooked rice. They stored the cooked rice at different temperatures, and results indicated that the higher the temperature, the faster the growth of the microorganism and the sooner the toxin was produced. The production of cereulide was strongly correlated with the growth of bacteria in boiled rice. The growth and toxin levels at 30 °C for 24 h were similar for boiled and fried rice. In the past, studies carried out with human volunteers [48,49,50] found a weak significance for symptoms when *B. cereus* cells were ingested.

A human dose response relationship has not been described for either the emetic or diarrhoeal toxin produced by *B. cereus*. Epidemiological evidence suggests that the majority of outbreaks worldwide due to *B. cereus* have been associated with concentrations higher than 10^5^ CFU/g in implicated foods [51,52,53].

### 3.1. Diarrhoeal Illness

The diarrhoeal syndrome is an example of a toxic infection. It is produced by enterotoxins. In the literature, the value quoted for the minimum infective dose for the diarrheal illness caused by *B. cereus* is generally higher than 10^5^ cells per gram [51,52,53]. Kramer and Gilbert [6] stated that the levels of *B. cereus* recovered from foods implicated in outbreaks of the diarrhoeal-type illness have always been within the range 5 × 10^5^ to 9.5 × 10^8^ CFU/g. The symptoms of *B. cereus* diarrheal type food intoxication include abdominal pain, watery diarrheal, rectal tenesmus, moderate nausea that may accompany diarrheal, seldom vomiting, and no fever [43]. Symptoms develop within 6–15 h and can persist for 24 h. This syndrome is rather mild and tends to mimic the symptoms of *Clostridium perfringens* food poisoning [54].

### 3.2. Emetic Illness

The emetic illness, where toxin is pre-formed in the food, requires a high cell concentration (10^5^ to 10^8^ CFU/g) to produce clinically significant amounts of toxin [54]. Heating foods before consumption might remove vegetative cells of *B. cereus* but it will not destroy the heat-stable emetic toxin. Infective dose, in the case of emetic illness, is not relevant, since the disease is intoxication dependent on the amount of toxin ingested. In an outbreak in Finland, a concentration of 1.6 μg/g of emetic toxin was found in the food, assuming that 300 g of food was consumed; the toxic dose could be as high as 450 μg/g [30]. In another outbreak in the Netherlands, concentrations of 0.03–13.3 μg/g of food were found [54]. The signs of *B. cereus* emetic type food poisoning include nausea, vomiting, and headaches, abdominal cramps, and/or diarrheal. The incubation period was estimated on 1 to 5 h after the consumption of food containing cereulide-heat-and gastric acid-resistant peptide. The symptoms of this illness mimic those of *Staphylococcus aureus* food poisoning [54].

## 4. Evaluation of Exposition to *B. cereus* in Rice

In general, foods are contaminated with *B. cereus* spores by soil. The number of cells in soil can range from 10^3^ to 10^5^ spores of *B. cereus* per gram [36,55]. Spores of *B. cereus* can develop biofilms due to its adhesive properties; in consequence, foods can be contaminated during processing when circulating by pipes, surfaces, or belts [56]. This microorganism frequently appears as a spore in ready-to-eat foods since the vegetative cells usually are destroyed by the thermal processes (cooking or frying). Storage of processed products without refrigeration or under temperature abuse, or the use of raw materials in complex foods allowing *B. cereus* spores to germinate and grow can represent a risk for consumers [34].

As for the development of new preservation technologies including combined processing (hurdle technologies) such as high hydrostatic pressures, pulsed electric fields, cooked chilled foods, among others, *B. cereus* has become an emerging risk, since these processes do not eliminate the spores and, in some circumstances, produce damaged vegetative cells that can grow at temperatures of the order of 10 °C or even lower.

Restaurants or catering facilities are the most frequent places where intoxication occurred. The main responsible for *B. cereus* proliferation in foods prepared in those facilities leading to poisoning were attributed, in many cases, to inaccurate refrigeration temperature and/or the delay before preparation and consumption of dishes [34].

Studies carried out on raw rice indicate that *B. cereus* spores are frequently isolated from this food, due to its ubiquity in nature. In fact, a prevalence of 100% was observed in 2010 in Argentina [57]. Likewise, in Colombia 244 samples of foods containing rice were analysed in different regions of the country and results showed that 11.92% of those foods have concentrations higher than 10^4^ CFU/g, concentrations that are considered of high risk [58].

Meals containing rice can also be a source of *B. cereus*. Studies carried out in restaurants of the United States and United Kingdom, where rice dishes were prepared, revealed that contamination can also occur after cooking, particularly through cross-contamination with spatulas used to mix rice during the cooking process [59]. In the UK, studies indicate that small restaurants pose a higher risk than large chain restaurants, owing to the poor training in hygienic practices, as well as the preparation of rice too early before being served [60].

Therefore, it is inevitable that, *B. cereus* due to its ubiquity, will be present in many raw materials. The contamination of the food during processing also requires the application of good hygienic practices and appropriate hygienic design of equipment as additional measures to control the contamination of products.

## 5. Control Measures

Control of *B. cereus* in rice and derivatives can be carried out at three different levels. First, there are the hygienic measures; second, the preservation processes in the production chain that would have the mission of destroying the microorganism and its spores; and third, control measures to slow down or inhibit the growth [61]. Table 1 summarizes the most important control measures described in literature.

The cleaning of equipment or machines where rice circulates in the industry is the first barrier to be applied to prevent the growth of the microorganism in surfaces or pipelines and the contamination of the rice by *B. cereus*. Its spores have the ability to adhere to stainless steel surfaces of industrial equipment; this favours the growth of the microorganism on these surfaces. The use of sodium hypochlorite and weak acids is recommended on pipes and other surfaces [34]. A hygienic design of the equipment is important to avoid dead areas where the microorganism’s spores can adhere and germinate.

The second barrier relies on the use of preservation procedures in the rice production chain that are capable of destroying vegetative cells and, where appropriate, bacterial spores. Heat treatment is the most common process for destroying spores and vegetative cells of microorganisms. However, *B. cereus* has a highly variable thermal resistance; in consequence, it is difficult to establish consistent pasteurization or cooking conditions. Fernandez et al. [40] reported for bacterial spores D-values at 90 °C between 3.99 min and 45 min for two strains of *B. cereus* isolated from vegetables. This difficulty is increased considering the great difference in thermal resistance between vegetative cells and spores. Byrne et al. [62] indicated that the *D*-values of *B. cereus* suggest that a mild cook of 70 °C for 12 s would achieve a 6 log reduction of *B. cereus* vegetative cells, while the equivalent reduction of *B. cereus* spores would be achieved after heating for 36 s at 105 °C in pork luncheon meat. Spores isolated from vegetables showed a D_105 °C_ value of 0.63 min in reference substrate (pH 7) [40]. This means that a F_105 °C_ value of 3.8 min is necessary to achieve a reduction of around 6 log; it should be taken into account that the normal cooking process of rice is carried out between 80 and 90 °C. Considering heat resistance data for very high heat resistant spores [40], heating food above 105 °C will be enough to kill *B. cereus* and protect the food from spoilage; nevertheless, only commercial sterilization ensures complete inactivation of *B. cereus* spores. Cooking, mild heat treatments, or regular pasteurization do not inactivate all *B. cereus* spores [36]. Mild heating processed such as indicated above can instead activate spores for germination and subsequent vegetative cell growth.

Several non-thermal technologies have been used to inactivate *B. cereus* spores, but their effect was variable. At ambient temperature, a pressure higher than 1000 MPa is required for inactivation bacterial spores [63]; in consequence, no *B. cereus* spores present in a rice dish can be inactivated at the lower ordinary industrial pressure treatments (600 MPa or less). Combined treatments can help in destroying the bacterial spores. Van Opstal et al. [64] studied the inactivation of *B. cereus* spores in milk by mild pressure and heat treatments. Results indicated that all strains were reduced more than 6 logs by the two-step treatment consisting on 30 min at 200 MPa/45 °C and 10 min of cooking at 60 °C. Pina-Perez et al. [65] reported that high hydrostatic pressure and natural antimicrobials were synergic against *B. cereus* vegetative cells in a mixture of liquid whole egg and skim milk.

Marco et al. [66] studied the joint effect of the antimicrobial olive powder and high hydrostatic pressure against *B. cereus* spores in a control substrate. The authors concluded that olive powder had an additive effect to the high hydrostatic pressure processing with storage temperature and could act as an additional control measure preventing the growth of microorganisms on products pasteurised by high hydrostatic pressure technologies or reducing the potential growth in the case of cold-chain break during the shelf life. Another non-thermal technology regarded as an effective decontamination method for rice raw material after the crop harvesting is the Cold Plasma [67]. Baia et al. [68] have studied the plasma technology to inactivate *B. cereus* spores; they achieved between 1.62–2.96 log CFU/mL reductions. Liao et al. (2020) [69] studied the application of plasma-activated water combined with mild heat for the decontamination of *B. cereus* spores in rice. The treatments achieved 1.54 and 2.12 log CFU/g reductions of *B. cereus* spores in rice after 60 min exposure.

The third control measure for *B. cereus* relies on avoiding or diminishing bacterial growth. The incidence of the *B. cereus* disease is linked to the food storage temperatures and the storage time before it is finally served. Freezing or cold storage of rice-based meals (temperature lower than 4 °C) is an important strategy to control *B. cereus* [61]. Growth of *B. cereus* can be reduced by increasing the generation time, increasing doubling times or the lag phase under refrigeration storage [70,71]. According to those studies, it appears that the main control measure avoiding growth of *B. cereus* in foods is the refrigeration at temperatures below 4 °C. Refrigeration can be combined with other methods to prevent microorganism’s growth. Modified atmosphere package, a carbon dioxide concentration higher than 40%, can prevent growth of *B. cereus* stored at a temperature lower than 8 °C [72,73]. At the same time, according to Andersson et al. [77], when the storage temperature was raised from 6 °C to 8 °C, growth of *B. cereus* was apparent, but after slight pH or water activity reductions, the growth of *B. cereus* was controlled at refrigeration temperatures higher than 4 °C [78]. Some food additives can be used alone or combined with other control measures against *B. cereus* in rice derivatives. Some bacteriocins such as nisin can inactivate *B. cereus* vegetative cells while essential oils-based antimicrobials such as carvacrol showed a limited effect [74,79,80]. Grande et al. [5] used enterocin AS-48 to inhibit toxicogenic *B. cereus* in rice-based foods. Inactivation of endospores was achieved by heating for 1 min at 90 °C in boiled rice or at 95 °C in rice-based gruel. Fernandes et al. [75] studied the antibacterial effects of chitosan on *B. cereus.* The use of chito-oligosaccharides alone against *B. cereus* spores was not enough to destroy a large number of cells. Ferrer et al. [76] concluded that olive powder could be used as an additional control measure in the case of cold chain break due to its effects on the lag phase *of B. cereus* vegetative cells.

In consequence, for full control of *B. cereus* concentration, it is essential to have a low initial concentration of *B. cereus* in raw materials and an adequate design of processing equipment. That should be followed by an effect preservation method and by effective cooling procedures to fast cool heat-treated foods, and storing the product below 4 °C. Those procedures will control the concentration of B. cereus up to acceptable levels for food safety.

## 6. Conclusions

*B. cereus* will grow in most foods under favourable pH (4.5 to 9.5), water activities (>0.93), and temperatures from 4 to 48 °C. Due to its ubiquity, its spores contaminate practically all categories of foods, rice and pasta meals being the most important source of *B. cereus* spores causing intoxication. Those spores have the ability to survive the treatments commonly given to rice and other carbohydrate-rich products. Rice cooking, the mild heat applications on rice refrigerated processed foods, or regular pasteurization, as well as many non-thermal technologies, do not inactivate all *B. cereus* spores. Only the commercial sterilization can assure the complete inactivation of spores. However, it is not always possible to provide a sterilization process to such foods, since in restaurants or in collective food preparation, sterilization is not used in the production of meals. Moreover, it is also necessary to consider the detrimental effect on the nutritional or sensory properties of sterilized foods.

The *B. cereus* concentration that consumers will face will depend on raw material contamination and preservation or processing technology, but is the multiplication of *B. cereus* in foods stored under abuse refrigeration temperature that is the main contributor to the risk for human health. Rapid cooling and subsequent refrigeration storage of heat treated foods is critical and should be carefully controlled to avoid the growth of vegetative cells during the cooling phase. Moreover, to complement refrigeration with slight reductions in pH or water activity of high-carbohydrate meals will prevent multiplication of *B. cereus* at refrigeration temperatures between 4 to 8 °C.

## Figures and Tables

**Table 1:** Some control measures for *Bacillus cereus* spores or vegetative cells.

Control Measure	Procedure	Treatment/Effect on *B. cereus*	Reference
Control initial microbial load	Use of sodium hypochlorite and weak acids on equipment	100 ppm sodium hypochlorite Weak acids at 30–40 °C for 20–30 min	[34]
Inactivation	Heat treatment	D-value (90 °C) 3.99–45 min 70 °C for 12 s, 6 log reduction (vegetative cells) 105 °C 36 s, 6 log reduction (spores)	[40,62]
	High Hydrostatic Pressure (HPP)	More than 1000 MPa	[63]
	Combined treatments	Mild heat and High Hydrostatic Pressure, between 100 and 600 MPa at 30 and 60 °C, 6 log inactivation	[64,65]
		Olive powder 2.5% and High Hydrostatic Pressure 500 MPa had additive effect	[66]
	Cold Plasma (CAP)	1.62–2.96 log CFU/mL reductions Plasma-activated water combined with mild heat, 1.5–2.12 log CFU/g reductions	[67,68,69]
Growth limitation or inhibition	Cold storage	Below 4 °C	[65,70,71]
		Carbon dioxide concentration higher than 40% can prevent growth of *B. cereus* stored at temperature lower than 8 °C	[72,73]
	Antimicrobials	Nisin, 500 IU/g	[74]
		Enterocin AS-48, 20–35 µg/ml	[5]
		Chitosan, 2.5% (*w*/*v*)	[75]
		Olive powder, 2.5% (*w*/*v*)	[76]
